# Investigations Touching the Use of Iodine

**Published:** 1864-01

**Authors:** 


### INVESTIGATIONS TOUCHING THE USE OF IODINE.

Dr. Rosenthal, assistant physician at the Vienna General Hospital, has published, in the *Wiener Med. Wochensch.*, a series of papers containing much original matter touching the therapeutic use of Iodine. The summing up is as follows :—

1. Large doses of iodide of potassium, combined with a small quantity of fluid, remain a long time in the economy; with large quantity of fluid, they are quickly washed away from the system and pass rapidly into the secretions and excretions. This circumstance should be carefully noticed.

2. When iodide of potassium is taken internally, it is found, not only in the urine, saliva, and other secretions, but also in the alvine evacuations, within from four to seven hours, whether the stools be aqueous or the reverse.

3. In the administration of iodide of iron, iodine is separated in considerable quantities and found with a large proportion of the iron in the urine. Fæcal matter contains much iron and a small amount of iodine. The same phenomena may be noticed when iodide of mercury is used.

4. Frictions with an ointment containing iodide of potassium upon sound skin will cause the iodine to be detached in the urine and saliva.

5. Iodine is found in the urine of those who take baths in which iodide of potassium is dissolved, even when the rectum and urethra are kept free from the action of the bath. This is proved by examining the urine, and by noting a large diminution of the iodine in the water used for the bath.

6. The intestinal mucous membrane takes of the iodine very energetically in the form of enemata, and this is the case even with very weak solutions of iodide of potassium.

7. Large doses of iodide of potassium, or small doses taken for a long time, are not well borne in certain pathological states of the economy; in fact, large doses of iodine, or concentrated solutions, are very prejudicial to the system.

